# Effectiveness of glycerin-oxalic acid strips and essential oils in controlling *Varroa destructor* in honeybee

**DOI:** 10.17221/94/2024-VETMED

**Published:** 2025-03-24

**Authors:** Plamen Rangelov Hristov, Ivaylo Stefanov Hristakov, Atanas Zdravkov Atanasov, Presiyan Antoniov Zhelyazkov

**Affiliations:** ^1^Agricultural Machinery Department, Agrarian and Industrial Faculty, University of Ruse, Ruse, Bulgaria; ^2^Institute of Animal Sciences, Kostinbrod, Bulgaria

**Keywords:** alternative treatment methods, apiculture health, mite infestation control, parasitic resistance

## Abstract

The decline in the bee colony strength due to high levels of *Varroa destructor* infestations necessitates the development of new control methods. This study evaluates the effectiveness of glycerin-oxalic acid strips and essential oils in managing *Varroa destructor*. The experiment was conducted in 2022 at the experimental apiary in Debelec, part of the Institute of Animal Husbandry in Kostinbrod. Six experimental groups, each treated with a different preparation, were compared to a control group of untreated colonies. Treatments included Beevital Hiveclean (20 ml per colony), Varro Red (2 ml per frame), ammonium nitrate strips (1.3 mm), glycerin-oxalic acid strips (1.3 mm), and glycerin-oxalic acid strips of paper-cellulose (2.3 mm) or cellulose-cotton (2.3 mm). The control group remained untreated. The glycerin-oxalic acid strips made of cellulose and cotton (2.3 mm) showed the highest efficacy, reducing the mite infestation by 17.79% compared to the control. The glycerin-oxalic acid strips of paper-cellulose (2.3 mm) were also effective, achieving a 17.05% reduction in mite levels. These results provide valuable insights for beekeepers seeking alternative and sustainable methods for controlling *Varroa destructor* infestations.

The life and development of a bee colony depend on various external and internal factors, including weather conditions, the presence of honey-bearing vegetation, agricultural practices, pesticides exposure, and parasites (Hristov et al. 2021). One of the most dangerous and widespread bee parasites is *Varroa destructor*, which is a primary factor in the weakening of the bees’ vitality and the collapse of entire colonies (Palmer 2019; Warner et al. 2024). Numerous studies (Ramsey et al. 2019; Yang and Cox-Foster 2005) have investigated the negative impacts of *Varroa destructor* on the bee health, specifically its role in transmitting viral diseases such as the Acute Bee Paralysis Virus (ABPV) (Le Conte et al. 2010). The population dynamics of honeybees and the infestation of honeybee colonies (*Apis mellifera*) by ABPV can be modelled and predicted using mathematical approaches (Ratti et al. 2013; Atanasov et al. 2022).

The active reproduction of *Varroa destructor* is synchronised with the reproductive cycle of bee colonies and the duration of brood rearing, depending on the climatic conditions of specific latitudes (Messan et al. 2020). In Bulgaria, this period coincides with the queen bee’s initial egg-laying at the end of winter and extends to the rearing of the last brood until late autumn. Investigating both the timing and mechanisms of the *Varroa destructor* reproduction is crucial for developing effective strategies to manage this parasite.

The primary methods for controlling *Varroa destructor* are categorised into chemical, biotechnological, and alternative approaches (Hristov 2021). One of the most common strategies involves the use of synthetic chemical compounds, primarily based on pyrethroids and organophosphates (Lambert et al. 2010; Haber et al. 2019; Bahreini et al. 2020). However, the excessive application of these compounds has been shown to promote resistance in *Varroa destructor*, reducing their efficacy over time (Stara et al. 2019; Roth et al. 2021). This issue can be partially mitigated by alternating synthetic treatments with different active substances. Additionally, some researchers have reported that chemical control methods contribute to the accumulation of harmful residue in wax combs and result in the contamination of the honey and pollen (Rinkevich 2020).

Biotechnological methods involve a series of beekeeping interventions within the nests of bee colonies. These methods include brood removal and/or the temporary cessation of all the sealed brood, followed by targeted mite treatment using established acaricidal preparations, or the use of “trap combs” for mite capture, with subsequent treatments (Hristov 2021). This approach is primarily suitable for a limited number of bee colonies, as it does not provide long-term control and can negatively impact the colony productivity (Parvanov 2016).

Considering the advantages and disadvantages of chemical and biotechnological methods, many researchers (Jack et al. 2021; Bava et al. 2023; Hazam et al. 2024) have proposed alternative strategies to combat *Varroa destructor*, focusing on biogenic preparations such as essential oils and organic acids. Treatments utilising essential oils have been shown to exhibit an acaricidal efficacy ranging from <30% to >70% (Hybl et al. 2021). According to Prouty et al. (2023), the highest efficacy of oxalic acid (OA) is achieved when applied via vaporisation at a dose 4 g every 5–7 days. Conversely, Bartlett et al. (2023) reported no evidence supporting the effectiveness of glycerol–oxalic acid mixtures delivered via paper towels for *Varroa destructor* control. Other studies have highlighted the potential negative effects of oxalic acid on drone bees, worker bees, and queen bees (Higes et al. 1999; Hernandez et al. 2007), as well as on the honey quality (Borsuk et al. 2012).

An interesting study was conducted by Kovacic et al. (2023) who investigated the effect of queen caging on the honey bee colonies’ post-treatment development and the optimal timing of the method application on honey production during the main summer nectar flow.

Various methods for controlling *Varroa destructor* have exhibited differing levels of effectiveness, influenced by factors such as the treatment type, frequency, local weather conditions, infestation severity, and the genetic resistance of bee populations. These variations indicate that the current control strategies may not be universally applicable, highlighting a need for region-specific solutions tailored to the unique environmental and biological conditions of different beekeeping areas. Although numerous studies have examined *Varroa destructor* management, a significant research gap remains concerning the efficacy of alternative treatments in specific regional contexts.

The objective of this study was to assess the effectiveness of glycerin-oxalic acid strips and the selected essential oils in controlling *Varroa destructor*, with a focus on adapting these methods to the specific conditions of the test region.

## MATERIAL AND METHODS

### Study apiary, preparations and biological material

The experiment was conducted in 2022 at the experimental apiary in Debelec, part of the Institute of Animal Sciences in Kostinbrod. The geographical coordinates of the experimental region are 42°58'50.88'' N and 25°44'43.97'' E. The bees used in the study belonged to the predominant race in Bulgaria, *Apis mellifera macedonica*. The experimental apiary in Debelec consisted of 80 bee colonies housed in Dadant-Blatt hives, each comprising 12 wooden frames made of softwood, with the following dimensions: length of 516 mm, width of 516 mm, height of 400 mm, and a wall thickness of 34 mm.

For the purposes of the experiment, a control group comprised of six bee colonies was established. These colonies were not subjected to any treatment during the experimental period. The experimental group included six distinct subgroups, each treated with ecological preparations containing essential oils, specifically Varro Red and Beevital Hiveclean, as well as the other subgroups with oxalic acid.

The subgroup treated with Varro Red comprised 13 bee colonies, while the subgroup treated with Beevital Hiveclean included 10 colonies. Another subgroup was treated with preparations containing oxalic acid, impregnated onto glycerin-oxalic acid strips made of paper-cellulose with thicknesses of 2.3 mm and 1.3 mm. The subgroup treated with glycerin-oxalic acid strips made of paper-cellulose with thicknesses of 1.3 mm initially consisted of seven colonies; however, two colonies were excluded during the season due to specific issues: one colony experienced a disruption in the queen bee identity, and another entered a swarming state.

Additionally, a subgroup treated with the glycerin-oxalic acid strips composed of cellulose and cotton (2.3 mm in thickness) included 12 colonies. A separate group of four colonies was established, treated with strips impregnated with a solution containing 0.5 parts by weight of ammonium nitrate and 1 part by weight of glycerin. This group was created for informational purposes only, as the data on the therapeutic effects were derived solely from empirical observations reported by some beekeepers.

The treatment of bee colonies with the strips from all the groups was carried out according to a scheme including placing an 18 cm long strip in each bee interspace. Each strip was folded over the top bar of the bee frame and lowered at both ends.

The treatment with the liquid preparations was carried out by dripping the preparation into the individual bee interspaces according to the manufacturer’s instructions.

For the liquid preparations, the treatment was performed by applying the preparation directly into the individual bee interspaces, following the manufacturer’s instructions to ensure consistent application.

The unequal number of bee colonies among the groups did not compromise the reliability of the statistical analyses, which included calculations of the mean values, standard errors of the mean, and the degrees of confidence in the observed differences. The methodology described by Lakin (1980) was employed to facilitate comparisons between groups with unequal sample sizes.

### Assessment of the main components of the examined preparations

The product marketed under the trade name Beevital Hiveclean is a liquid used for treating bees in the hive. The primary ingredients in 1 litre of the product include a propolis solution, essential oils, E-150d, 42 g of oxalic acid dihydrate, 4.88 g of formic acid, and citric acid stabilised in a sugar solution. Depending on the strength of the colony, the recommended dosage per colony ranges from 10 ml to 20 ml. For the colonies in this study, a dosage of 15 ml per colony was used.

Varro red produced by the Balmina company was also used to treat the bee colonies. The preparation is an organic liquid product for fighting mites. The primary ingredients in 1 litre of the preparation include: formic acid (4.250 mg l^–1^), Ponceou 4R (E124) (200 mg l^–1^), citric acid (E330) (50 mg l^–1^), aromatic extracts (14 mg l^–1^), sucrose (600 mg l^–1^), demineralised oxidane (600 mg l^–1^). The recommended treatment dose is 2 ml per frame.

Two other experimental groups of bees were treated with glycerin-oxalic strips made of paper-cellulose and glycerin-oxalic strips made of cellulose and cotton. The strips had a thickness of 2.3 mm, a width of 2.5 cm and a length 40 cm. A separate group was treated with glycerin-oxalic strips made of paper-cellulose with a thickness of 1.3 mm. The strips were impregnated with a solution containing 67% pharmaceutical vegetable glycerin and 33% high purity oxalic acid. To study the therapeutic effect of ammonium nitrate, cardboard strips with a thickness of 2.3 mm, a width of 2.5 cm and a length of 45 cm were prepared. These strips were impregnated with a working solution containing 0.5% ammonium nitrate and 1 part by weight of glycerin.

### Method of treatment of the bee colonies

During the active season (April 1, 2022, to July 31, 2022), the colonies were deliberately infested by introducing a single brood comb containing *Varroa destructor*-infected drone brood sourced from colonies not included in the experiment.

Twenty days prior to the first treatment (April 1, 2022), colonies were equalised for the brood size and honey stores. This procedure ensured that all the colonies began the experimental period under comparable conditions in terms of the population size, brood area, and other relevant parameters. Equalisation was conducted simultaneously for all the experimental groups to standardise the baseline conditions and to minimise the variability among the colonies at the start of the study.

The time of the equalisation of the colony strength and dates of all the performed treatments is shown in Table 1.

**Table 1:** Dates of the equalisation of the colony strength and treatment and the results reporting dates

Medicinal product	Dates of equalisation of colony strength	Treatment dates	Results reporting dates
Beevital Hiveclean	10.03.2022	04.09.2022	26.10.2022
Varro red	10.03.2022	01.04.2022	26.10.2022
Varro red	10.03.2022	07.04.2022	26.10.2022
Varro red	10.03.2022	14.04.2022	26.10.2022
Varro red	10.03.2022	29.08.2022	26.10.2022
Varro red	10.03.2022	05.09.2022	26.10.2022
Glycerin-oxalic strips, Greece production (1.3 mm)	10.03.2022	09.08.2022	26.10.2022
Ammonium nitrate	10.03.2022	09.08.2022	26.10.2022
Glycerin-oxalic strips of paper-cellulose (2.3 mm)	10.03.2022	09.08.2022	26.10.2022
Glycerin-oxalic strips of cellulose and cotton (2.3 mm)	10.03.2022	09.08.2022	26.10.2022

The treatment with the Beevital Hiveclean preparation was carried out once on September 4, 2022 between 19:00 h and 20:00 h, after the bees from the colony had returned to the hive. The air temperature during the treatment was 14 °C, which fell within the recommended range of 5 °C and 25 °C specified in the product instructions. The therapeutic efficacy of the treatment was evaluated on October 26, 2022. In the spring, a triple treatment with Varro red was carried out. The first treatment was on April 1, 2022. The interval between separate treatments was on seven days between 19:00 and 20:00, when the bees of the colony had returned to the hive at an air temperature of 14 °C. In the fall, a two-time treatment with Varro red was carried out. The therapeutic efficacy of the treatment was evaluated on October 26, 2022.

**Figure 1:**
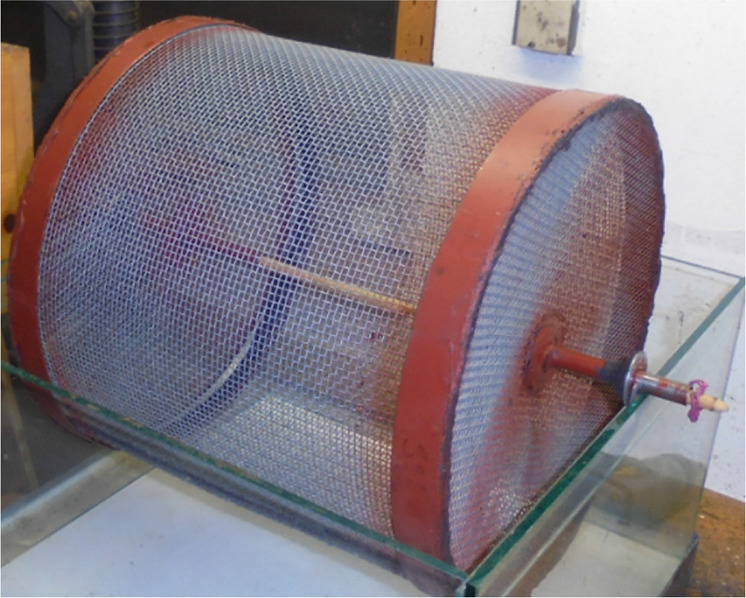
Mesh container for collecting bees

The placement of glycerin-oxalic strips began on August 9, 2022. The therapeutic effect of the treatment was assessed on October 26, 2022. On this date, the amount of the sealed brood in the bee colonies was, on average, between 1–3 dm^2^. The area of the capped brood was determined using the method proposed by Lazarov and Dineva (2022). After equalisation, no combs were shared between the colonies to ensure no further mechanical transfer of *Varroa destructor*. The collection of bees from the individual colonies was conducted according to the methodology of Gaidar (2011). From each bee colony of the control and experimental groups, 300–400 bees were taken, collected in a mesh container (Figure 1) by sweeping from the frames with a brood. The collected bees were anesthetised with alcohol, then soaked with detergent, mixing with the bees, after which the bees were thoroughly washed with a high-pressure water jet and passed through a fine strainer. The mites and bees separated from the sample were counted and their percentage was calculated.

### Statistical analyses

For the statistical processing of the data obtained, the software STATGRAPHICS Plus v5 (Statistical Graphics Corporation, Warrenton, VA, USA) was used. To evaluate the effectiveness of the preparations used against *V. destructor* mites and adult bees, they were processed by one-way analysis of variance (ANOVA).

## RESULTS AND DISCUSSION

The results indicate that most of the tested preparations exhibited acaricidal effects, as shown in Table 2 and Table 3. However, the ammonium nitrate strips (1.3 mm thick) did not demonstrate a therapeutic effect. The highest infestation rate, 51.78%, was observed in the colonies treated with these strips, representing a 33.66% higher degree of parasitism compared to the control group. It can be hypothesised that some unaccounted factors, such as variations in the colonies’ cleaning behaviour, may have influenced the results.

**Table 2:** Degree of infestation of bee colonies treated with glycerin-oxalic strips and ammonium nitrate

Types of treatments	Variant	Number of bees in the sample	Number of *Varroa destructor*	Level of infestation (%)
Control	1	343	63	18.37
	1	405	14	3.46
	1	251	14	5.58
	1	215	176	81.86
	1	315	34	10.79
	1	287	28	9.76
Ʃ	–	1 816	329	18.12
Statistical indicators	–	*n* = 6	σ = **±**29.95	Sx^2^ = **±**178.75
Glycerin-oxalic strips, Greece production (1.3 mm)	2	232	1	0.43
	2	163	1	0.61
	2	248	5	2.02
	2	265	2	0.75
	2	317	27	8.52
Ʃ	–	1 225	43	3.5
Statistical indicators	–	*n* = 5	σ = **±**3.44	Sx^2^ = **±**2.96
Ammonium nitrate	3	196	121	61.73
	3	216	207	95.83
	3	283	103	36.40
	3	317	93	29.34
Ʃ	–	1 012	524	51.78
Statistical indicators	–	*n* = 4	σ = **±**45.01	Sx^2^ = **±**506.70
Glycerin-oxalic strips of paper-cellulose (2.3 mm)	4	304	2	0.66
	4	287	0	0.00
	4	290	1	0.34
	4	236	0	0.00
	4	171	0	0.00
	4	372	4	1.08
	4	247	9	3.64
	4	157	3	1.91
	4	361	8	2.22
	4	270	1	0.37
	4	240	1	0.42
	4	244	5	2.05
Ʃ	–	3 179	34	1.07
Statistical indicators	–	*n* = 12	σ = **±**1.154	Sx^2^ = **±**0.12
Glycerin-oxalic strips of cellulose and cotton (2.3 mm)	5	183	0	0.00
	5	191	0	0.00
	5	181	0	0.00
	5	280	3	1.07
	5	115	0	0.00
	5	151	1	0.66
	5	97	0	0.00
Ʃ	–	1 198	4	0.33
Statistical indicators	–	*n* = 7	σ = **±**0.44	Sx^2^ = **±**0.032

**Table 3:** Degree of infestation of the bee colonies treated with the ecological preparations containing essential oils

Types of treatments	Variant	Number of bees in the sample	Number of *Varroa destructor*	Level of infestation (%)
Varro red	6	168	9	5.36
	6	182	5	2.75
	6	239	20	8.37
	6	167	1	0.60
	6	193	1	0.52
	6	212	2	0.94
	6	165	1	0.61
	6	197	1	0.51
	6	199	3	1.51
	6	139	4	2.88
	6	216	9	4.17
	6	179	3	1.68
	6	122	3	2.46
Ʃ	–	2 378	62	0.26
Statistical indicators	–	*n* = 13	σ = **±**2.32	Sx^2^ = **±**0.45
Beevital Hiveclean	7	220	8	3.64
	7	228	1	0.44
	7	205	9	4.39
	7	213	10	4.69
	7	198	4	2.02
	7	170	21	12.35
	7	225	17	7.56
	7	247	33	13.36
	7	228	5	2.19
	7	203	16	7.88
Ʃ	–	2 137	124	5.80
Statistical indicators	–	*n* = 10	σ = **±**9.08	Sx^2^ = **±**8.23

The treatment of the bee colonies with the glycerin-oxalic acid strips (1.3 mm in thickness), resulted in a 14.62% lower infestation rate compared to the control group; however, this difference was not statistically significant. When the colonies were treated with the glycerin-oxalic acid strips made of paper-cellulose (2.3 mm in thickness), a favourable therapeutic effect was observed, although some individual colonies exhibited a reduced response. Similar instances of variability, with more pronounced exceptions affecting approximately 20% of samples, were reported in previous studies by Hristov and Ivanov (2021).

Very favourable results were obtained from the treatment with glycerin-oxalic acid strips made of cellulose and cotton (2.3 mm in thickness), where the infestation level was only 0.33%. A comparison between the treatment using glycerin-oxalic acid strips, 1.3 mm in thickness, and those made of paper-cellulose (2.3 mm in thickness), reveals that the infestation level with the 1.3 mm strips was 2.43% higher than with the paper-cellulose strips; however, this difference is not statistically significant. Both treatment methods exhibited therapeutic effects, but the efficacy is enhanced with thicker strips. Thinner healing strips can be more easily dislodged from the hives, and their therapeutic effect is often insufficient. A notable concern is that normally strong colonies can remove them from the hive within 10 to 15 days after placement. Upon inspection, only remnants of partially gnawed tape were found in the hives.

On the other hand, the use of glycerin-oxalic acid strips made of paper-cellulose (2.3 mm in thickness), and glycerin-oxalic acid strips made of cellulose and cotton, also (2.3 mm in thickness), resulted in a difference of 0.714% in the level of infestation with the glycerin-oxalic acid strips made of cellulose and cotton. The confidence level was low (*P* ≥ 0.95); however, a better therapeutic effect was observed with the glycerin-oxalic acid strips made of cellulose and cotton, which is attributed to the superior absorbent capacity of the cotton base and the extended period during which the strips remained in the hive.

Similar studies on the therapeutic effect of glycerin-oxalic acid mixtures delivered via paper towels for controlling *Varroa destructor* were reported by Bartlett et al. (2023), who found a negligible therapeutic effect. In contrast, our research demonstrated a significant therapeutic effect, emphasising the importance of the substrate upon which the glycerin-oxalic acid mixture is applied and the duration of its presence in the bee colony. These findings suggest that the effectiveness of such treatments is strongly influenced by these factors.

The results presented in Table 3 show that the application of the Varro Red preparation resulted in an average infestation level that was 0.64% lower than that observed with the glycerin-oxalic acid strips (1.3 mm in thickness) and 2.53% higher than the treatment with glycerin-oxalic acid strips made of cellulose and cotton (2.3 mm in thickness). The comparison between the glycerin-oxalic acid strips made of paper-cellulose (2.3 mm in thickness) and the preparation Varro Red indicates that the infestation level is 1.71% lower in favour of the glycerin-oxalic acid strips. However, the difference was not statistically significant, suggesting that both treatments exhibited a therapeutic effect.

In a comparison between the preparations Varro Red and Beevital Hiveclean, it was found that the level of infestation with Beevital Hiveclean is 2.94% higher than with Varro Red, with a highly significant difference of *P* ≥ 0.999, confirming the superior therapeutic efficacy of Varro Red. The application of both preparations demonstrated acaricidal effects in controlling mite populations in the colony; however, the residual mite count after the treatment remained relatively high. Compared to the control group, the use of Varro Red resulted in a 15.86% lower level of infestation, while the application of Beevital Hiveclean resulted in a 12.32% reduction.

Neither preparation was studied for its effects on honey bees. In addition to our research, the acaricidal effects of different essential oils have been confirmed in several studies.

Begna et al. (2023) assessed the toxicity of essential oil extracts from *Eucalyptus globulus* Labill., *Rosmarinus officinalis* L., and *Trachyspermum ammi* (L.) Sprague against the honey bee mite, *Varroa destructor*. Conversely, studies by Narciso et al. (2024) reported no acaricidal effects when using cinnamon and oregano essential oils, as well as a mixed fruit cocktail juice. However, these studies confirm the safety of the oils used on honey bees. Alsaadi et al. (2024) achieved efficacy rates of 70.0% and 53.3% compared to control groups in treatments using nine essential oils extracted from plant materials.

The results of this study demonstrate that glycerin-oxalic acid strips, particularly those made from cellulose and cotton (2.3 mm in thickness), show a significant reduction in *Varroa destructor* infestation levels, outperforming the other treatments, including paper-cellulose strips and essential oils. These findings suggest that glycerin-oxalic acid strips offer an effective alternative mite control, particularly in regions with comparable environmental and beekeeping conditions.

The study also underscores the importance of considering local factors – such as the climate, colony infestation levels, and bee breed resistance – when selecting mite control strategies. Future research should aim to refine these methods across various regions to improve their efficacy and sustainability in diverse beekeeping practices.

### Limitations of glycerin-oxalic acid strips and essential oils

Effectiveness variability: The effectiveness of glycerin-oxalic acid strips and essential oils can vary significantly based on local environmental conditions, such as the temperature and humidity, which may affect the distribution and action of the treatments within the hive.

Impact on bees: While generally considered safer than synthetic chemicals, the improper application or overdosing of glycerin-oxalic acid and essential oils can have harmful effects on the bee health, including stress and reduced colony strength.

Application complexity: The application of glycerin-oxalic acid strips and essential oils may require specific techniques and frequency, which can be labour-intensive and may not be feasible for all beekeepers.

Limited long-term efficacy: Glycerin-oxalic acid and essential oils are often most effective as part of an integrated pest management strategy, rather than as standalone solutions. This necessitates a more complex management approach that may not always be practical.
